# Oxytocin Receptor Gene Polymorphisms Are Associated with Human Directed Social Behavior in Dogs *(Canis familiaris)*

**DOI:** 10.1371/journal.pone.0083993

**Published:** 2014-01-15

**Authors:** Anna Kis, Melinda Bence, Gabriella Lakatos, Enikő Pergel, Borbála Turcsán, Jolanda Pluijmakers, Judit Vas, Zsuzsanna Elek, Ildikó Brúder, Levente Földi, Mária Sasvári-Székely, Ádám Miklósi, Zsolt Rónai, Enikő Kubinyi

**Affiliations:** 1 Department of Ethology, Eötvös Loránd University, Budapest, Hungary; 2 Research Centre for Natural Sciences, Institute of Cognitive Neuroscience and Psychology, Hungarian Academy of Sciences, Budapest, Hungary; 3 Comparative Ethology Research Group, MTA-ELTE, Budapest, Hungary; 4 Department of Medical Chemistry, Molecular Biology and Pathobiochemistry, Semmelweis University, Budapest, Hungary; 5 Department of Ethology and Animal Welfare, Catholic University College Ghent, Brugge, Belgium; 6 Department of Animal and Aquacultural Sciences, Norwegian University of Life Sciences, Ås, Norway; Institut d'Investigacions Biomèdiques August Pi i Sunyer, Spain

## Abstract

The oxytocin system has a crucial role in human sociality; several results prove that polymorphisms of the oxytocin receptor gene are related to complex social behaviors in humans. Dogs' parallel evolution with humans and their adaptation to the human environment has made them a useful species to model human social interactions. Previous research indicates that dogs are eligible models for behavioral genetic research, as well. Based on these previous findings, our research investigated associations between human directed social behaviors and two newly described (−212AG, 19131AG) and one known (rs8679684) single nucleotide polymorphisms (SNPs) in the regulatory regions (5′ and 3′ UTR) of the oxytocin receptor gene in German Shepherd (N = 104) and Border Collie (N = 103) dogs. Dogs' behavior traits have been estimated in a newly developed test series consisting of five episodes: Greeting by a stranger, Separation from the owner, Problem solving, Threatening approach, Hiding of the owner. Buccal samples were collected and DNA was isolated using standard protocols. SNPs in the 3′ and 5′ UTR regions were analyzed by polymerase chain reaction based techniques followed by subsequent electrophoresis analysis. The gene–behavior association analysis suggests that oxytocin receptor gene polymorphisms have an impact in both breeds on (i) proximity seeking towards an unfamiliar person, as well as their owner, and on (ii) how friendly dogs behave towards strangers, although the mediating molecular regulatory mechanisms are yet unknown. Based on these results, we conclude that similarly to humans, the social behavior of dogs towards humans is influenced by the oxytocin system.

## Introduction

Dogs have been living in the human environment for several millennia [1]. During this cohabitation many human-like social skills emerged, such as attachment to the owner [2], [3], following of human referential gestures [4], [5], sensitivity to human ostensive cues [6], [7] that were presumably facilitated by the process of domestication [8], [9]. This particular domestication history and the special socio-cognitive skills are the reason why dogs became a widely used model species, also in behavioral genetics [10], [11]. Dogs' special bond to humans, their complex human-analogue social behavior and the fact that their genome has been sequenced [12] make them ideal candidates for this kind of research. A further advantage is that several human psychiatric disorders have an analogue in dogs [13], [14], while in the traditionally used rodent models these conditions have to be induced artificially.

Another line of research has identified several underlying neural, hormonal and genetic mechanisms that contribute to human sociality. Special attention has been devoted to the oxytocin system [15]. The central actions of oxytocin include regulating reproductive behavior, mother-offspring attachment [16] and social memory [17]. Due to its peripheral effects oxytocin is mostly known as female hormone, but it affects socio-cognitive capacities in both women [18] and men [19], although there might be differential effects [20]. Moreover the oxytocin system is involved in several neurological disorders such as autism, depression and social anxieties [21].

The oxytocin system is evolutionarily conserved, both the hormone and its receptor are present in mammals and other taxa [16], [22]. However variations caused by genetic polymorphisms might modulate the function of this complex system (e.g. humans: [23], prairie voles [24]). Polymorphisms in the oxytocin receptor (OXTR) gene have been shown to influence human social behaviors such as attachment [25], [26] or empathy [27].

It has already been shown that the similarity between the human and the dog OXTR gene is high [28]. The human peptide is composed of 389 amino acids, while the dog version contains 384 amino acids. Twenty-six locations contain different amino acids, but eight of these are similar in chemical properties (polarity, acidity).

So far no information on the dog OXTR gene polymorphisms are available thus the role of these polymorphisms in regulating behavior is also unexplored. Our aim in the current exploratory study was threefold: (1) In Study 1 we developed a test series that quantifies human-directed social behavior in dogs; (2) In Study 2 we identified new markers in the dog's oxytocin receptor gene; (3) In Study 3 we searched for preliminary evidence of possible associations between human-directed social behavior and OXTR gene polymorphisms in two dog breeds (German Shepherds and Border Collies).

### Ethics Statement

Non-invasive studies on dogs are currently allowed to be done without any special permission in Hungary by the University Institutional Animal Care and Use Committee (UIACUC, Eötvös Loránd University, Hungary). The currently operating Hungarian law “*1998. évi XXVIII. Törvény”* – the Animal Protection Act – defines experiments on animals in the 9^th^ point of its 3^rd^ paragraph (3. §/9.). According to the corresponding definition by law, our non-invasive observational study is not considered as an animal experiment. The owners volunteered to participate and approved of the genetic analyses and behavioral testing of their dogs.

All data (including video recordings of the behavioral tests, behavioral coding and genotype data) is stored in the Department of Ethology, Eötvös University, Budapest and is available upon request. The video protocol of the behavioral test is available in the Comparative Mind Database *(* http://www.cmdbase.org/web/guest/play/-/videoplayer/222 *)*. Genotypic data is available in the NCBI dbGaP data repository (http://www.ncbi.nlm.nih.gov/gap).

## Study 1

### Background

Although the dog has been proved to be an ideal behavioral genetic model species [29], gene × behavior association studies using individual phenotyping are scarce. The most widely used method relies on breed stereotypes provided by experts such as dog-trainers [30], [31]. In Study 1A our aim was to develop in a group of pet German Shepherd dogs a test series measuring human-directed social behavior. We intended to find behavioral scales with high internal consistency and inter-observer reliability that is crucial for measuring behavioral traits [32]. In order to further validate the behavioral scales, in Study 1B we applied the same test on Border Collies of two different countries in order to see whether the same behavioral scales are applicable.

### Method

Study 1A. Subjects were 104 privately owned adult (>1 year; mean age±SD: 3.88±2.55) German Shepherd dogs (male/female: 58/46). None of the subjects were closely related, i.e. littermate and parent-offspring relationships were excluded. In order to describe and validate a test series measuring human-directed social behavior subjects participated in a standard test series conducted outdoors, comprised of the following episodes:

1. Greeting: the owner (O) stands motionless next to the dog and holds the leash. An unfamiliar experimenter (E) approaches them in a friendly way. E stops out of reach of the leash and waits for 3 seconds. If the dog is not aggressive, E steps next to the dog then pets the dog's head and back. Then E steps away and waits for another 3 seconds.
2. Separation from the owner: the dog is tethered to a tree on a leash, while O is hiding behind an object (e.g. a big tree) which is at 5–6 m from the dog, and blocks the dog from seeing the owner. After 1 min has elapsed E approaches the dog and greets it (see description at Test 1: Greeting). Then E initiates play with a tug for 30 seconds. Then E steps back to the camera. After 1 minute has elapsed, E asks the O to come back and greet the dog (see description at Test 1: Greeting). Afterwards O initiates play with a tug.
3. Problem solving: E puts a piece of food into a small cage that can be retrieved by rolling over the cage. The O stands 1 m in front of the cage, holds the leash of the dog and is not allowed to speak or gesticulate. The dog has 1 minute to manipulate the cage. Trial ends when the dog gets the food, or after the 1 minute elapsed (in which case the E gives the food to the dog). This trial is repeated once.
4. Threatening stranger: O stands motionless next to the dog and holds the leash. E steps back 10 meters, and approaches the dog slowly, by leaning forward her upper body and staring at the eyes of the dog. E stops approaching when the dog shows signs of aggression, severe fear or when she reached the dog. Finally, the E steps back to the starting point, crouches, and asks the O to let the dog free. Then she starts to call the dog in a friendly way.
5. Hiding: E takes the dog on the leash, meanwhile the O is asked to hide behind a large tree 15–20 m away from the dog. After 30 seconds, independently from the orientation of the dog, the E releases the dog and says “Go!”. If the dog does not start to move immediately, the E once pushes it gently by touching the rear end of the dog. If the dog does not start to move within 5 seconds then the E asks the owner to call the dog.

Behavioral variables were coded on a 0–3 scale in each episode (**Table 1**).

**Table 1:** Behavioral variables coded in each test.

Episode	Variable (Abbreviation)	Definition 0–3 score			
*Greeting*	**Latency of approaching E** (GrApp)	0 s	1–5 s	5–15 s	Does not approach
*Greeting*	**Latency of following E** (GrFoll)	0 s	1–5 s	5–15 s	Does not follow
*Separation*	**Duration of orientation to O (1)** (SepOriO1)	0%	1–50%	51–99%	100%
*Separation*	**Latency of approaching E** (SepAppE)	0 s	1–5 s	5–15 s	Does not approach
*Separation*	**Latency of following E** (SepFollE)	0 s	1–5 s	5–15 s	Does not follow
*Separation*	**Duration of orientation to O (2)** (SepOriO2)	0%	1–50%	51–99%	100%
*Separation*	**Latency of approaching O** (SepAppO)	0 s	1–5 s	5–15 s	Does not approach
*Separation*	**Latency of following O** (SepFollO)	0 s	1–5 s	5–15 s	Does not follow
*Separation*	**Duration of playing with the O** (SepPlayO)	0%	1–50%	51–99%	100%
*Problem solving*	**Number of orientations to O (1)** (ProblOriO1)	0%	1–50%	51–99%	100%
*Problem solving*	**Number of orientations to E (1)** (ProblOriE1)	0%	1–50%	51–99%	100%
*Problem solving*	**Number of orientations to O (2)** (ProblOriO2)	0%	1–50%	51–99%	100%
*Problem solving*	**Number of orientations to E (2)** (ProblOriE2)	0%	1–50%	51–99%	100%
*Threatening approach*	**Jumping ups** (ThreJump)	0	1	2	≥3
*Threatening approach*	**Friendly–Aggressive (subjective score)** (ThreFrieAgg)	Aggressive	Neutral	2	Friendly
*Threatening approach*	**Latency of approaching during calling** (ThreCall)	0 s	<10 sec	10–30 sec	Does not approach
*Hiding*	**Latency of approaching O** (HideAppO)	0 s	1–5 s	5–15 s	Does not approach
*Hiding*					

Definitions for the 0–3 scores of each behavioral variable coded during the five tests are provided.

Study 1B. We applied the same test series to N = 103 adult (>1 year; mean age ±SD: 4.28±2.74) Border Collies (male/female: 46/57) from two countries (59 from Hungary and 44 from Belgium) in order to test if the test series is valid for a different breed and for different countries.

#### Statistical analysis

First, a Principal Component Analysis was carried out on all behavioral variables coded for German Shepherds and behavioral scales were created. Internal consistency of the scales was characterized by Cronbach's Alpha values. In order to check inter-rater reliability 20 test videos were coded by two independent raters and interclass correlation [33] was calculated between them for each of the four behavioral scales.

Second, behavior of all subjects was characterized by calculating the behavioral scales based on the structure obtained in German Shepherd dogs, and the two populations of Border Collies as well as the two different breeds were compared (independent samples t-tests).

### Results

The Principal Component Analysis of German Shepherd dog behavioral data resulted in four behavioral scales (**Table 2**):

**Table 2:** Factor loads of the different variables on each behavioral scale.

	Proximity seeking	Reaction to separation from owner	Friendliness	Looking at humans
SepAppE	**0.812**			
SepFollE	**0.766**			
GrApp	**0.511**			
SepPlayO	**0.447**			
ThreCall	**0.445**			
SepFollO	**0.424**			
SepOriO2		**0.880**		
SepOriO1		**0.857**		
HideAppO	0.237	**0.643**		0.207
ProblOriE2			**0.884**	
ThreFrieAgg			**0.795**	
ThreJump			**0.550**	
ProblOriO2				**0.829**
ProblOriO1				**0.777**
ProblOriE1		0.207		**0.557**

Behavioral variables that were related to any of the four scales according to the Principal Component Analysis and their factor loads are shown; values <0.2 are suppressed for the sake of clarity.

Proximity seeking is related to how willingly the dog approaches and interacts with both the owner and a stranger and is composed of the following variables: *Greeting* Approach E; *Separation* Approach E, Follow E, Follow O, Play with O; *Threatening approach* Call (Cronbach α = 0.628).

Reaction to separation from owner is related to how intensely the dog shows owner-directed behaviors when left alone or with a stranger and is composed of the following variables: *Separation* Orientation to O (1), Orientation to O (2); *Hiding* Approach O (Cronbach α = 0.753).

Friendliness is related to the dog's behavior in reaction to a threatening stranger and to a passive stranger while facing a problem box and is composed of the following variables: *Problem solving* Orientation to E (2); *Threatening approach* Friendly–Aggressive, Jumping ups (Cronbach α = 0.525).

Looking at humans is related to the number of times the dog looks at the passive owner and stranger while facing a problem box and is composed of the following variables: *Problem solving* Orientation to O (1), Orientation to O (2), Orientation to E (1) (Cronbach α = 0.611).

According to the interclass correlations, all four scales are highly reliable between raters: Proximity seeking: r = 0.961, p<0.001; Reaction to separation from owner: r = 0.806, p<0.001; Friendliness: r = 0.861, p<0.001; Looking at humans: r = 0.943, p<0.001.

Internal consistency of the behavioral scales was also high for Border Collies: Proximity seeking: α = 0.692; Reaction to separation from owner: α = 0.502; Friendliness: α = 0.695; Looking at humans: α = 0.739, thus validating the behavioral scales on an independent sample.

Behavior of Border Collies from the two countries did not differ with respect to Proximity seeking (t_(101)_ = 1.758, p = 0.082), Reaction to separation from owner (t_(101)_ = 0.528, p = 0.598) and Friendliness (t_(101)_ = 0.354, p = 0.724), although Belgian dogs scored higher for Looking at humans (t_(101)_ = 3.597, p = 0.001).

Behavior of German Shepherds and Border Collies on the other hand was considerably different with Border Collies showing more Proximity seeking (t_(213)_ = 3.240, p = 0.001), a weaker Reaction to separation from owner (t_(213)_ = 6.493, p<0.001), less Friendliness (t_(213)_ = 2.561, p = 0.011) and more Looking at humans (t_(213)_ = 2.540, p = 0.012).

## Study 2

### Background

Polymorphisms in the OXTR gene have been implicated in the regulation of a wide range of human social behaviors [25]–[27]. Furthermore it has already been shown that the similarity between the human and the dog OXTR gene is high [28]. However no information is yet available on the dog OXTR gene polymorphisms. Thus the aim of Study 2 was to identify SNPs in the OXTR gene of German Shepherds and Border Collies, as well as to characterize these two breeds for allele frequencies, Hardy-Weinberg equilibrium and Linkage Disequilibrium.

### Method

#### Sequencing

Buccal samples were collected from all dogs participating in Study 1 in a non-invasive way, with cotton swabs from the inner surface of the cheek. Genomic DNA was extracted from buccal swabs using standard protocol. The sequence of the dog OXTR gene was obtained from the GenBank (http://www.ncbi.nlm.nih.gov/) and Ensembl (http://www.ensembl.org/) databases, accession numbers were as follows: NC_006602 and ENSCAFG00000005553 in the two databases, respectively. The sequence of protein coding and the surrounding regulatory regions (582 bp of 5′ flanking region and 585 bp of 3′ flanking region) of dog OXTR gene was determined by polymerase chain reaction (PCR) amplification and subsequent direct sequencing performed on 3-3 individuals of five different dog breeds (German Shepherd, Siberian Husky, Beagle, Border Collie, Golden Retriever), respectively. PCR primers were designed by NCBI/Primer-Blast (http://www.ncbi.nlm.nih.gov/tools/primer-blast/). The Qiagen Hot-StarTaq polymerase kit was used for PCR amplification. The reaction mixture contained 1 µM of each primer (Table 3), approximately 5 ng of DNA template, 200 µM dNTP, 0.025 U HotStarTaq DNA polymerase, 1× buffer, and 1× Q-solution supplied together with the enzyme. The PCR cycle consisted of an initial denaturation at 95°C for 15 minutes, 35 cycles of 1-min denaturation at 95°C, 1-min annealing at various temperatures (Table 3), a 1-min extension at 72°C, and a 10-min final extension at 72°C. The PCR reaction was performed in a total volume of 30 µl. The obtained PCR products were cleaned by Wizard SV Gel and PCR Clean-Up System (Promega, A9282) and sequenced in both forward and reverse directions with the same PCR primers (Macrogen Europe). SNPs were identified by aligning and comparing the sequence data with an Internet program (http://www.genome.jp/tools/clustalw/).

**Table 3:** Sequencing primers and annealing temperatures used for PCR amplification of dog OXTR gene regions.

	Primer	Sequence (5′-3′)	T_A_ (°C)
5′ flanking region	Forward	GGATGGTGCTGAGCGGGGGA	62
	Reverse	GGCCGTGCGGTTGCCCT	62
exon 1 5′ region	Forward	GTGAGCGCTCGGTCTTCTC	56
	Reverse	CAGCGGCTGGCAGATGG	56
exon 1 3′ region	Forward	CATGTTCGCCTCCACCTACC	56
	Reverse	GCCCCGCTCGCTACCTT	56
exon 2	Forward	GAAAGGCCATTCTCAGGAAA	52
	Reverse	CCCCCATCATCTTCTACCA	52
3′ flanking region	Forward	TAGACAGTCCGCCCCTTGGTGG	58
	Reverse	CACCTTCTGACATGCTGGTGCCC	58

#### Genotyping

From the three polymorphisms identified (see Results below) −212AG SNP was genotyped by PCR-RFLP method. PCR amplification was performed as described above using 5′-CCA TTG GAA TCC GCC CCC T-3′ forward and 5′-CAC CAC CAG GTC GGC TAT G-3′ reverse primers. Annealing temperature was 56°C and total reaction volume was 10 µl. PCR products were incubated for 3 h at 37°C in a restriction enzyme mixture containing 0.5 U/µl Hpy99I restriction enzyme (NEB), 1× BSA and 1× NEB4 buffer. Total reaction volume was 16 µl. The digested PCR products were analyzed by conventional submarine agarose gel electrophoresis (Biocenter, Szeged, Hungary), using 2.5% agarose gel and visualized by ethidium bromide staining.

19131AG and rs8679684 SNPs were genotyped by real-time PCR using sequence specific TaqMan probes with minor groove binding (MGB) quencher. Primers were designed by Primer Express 3.0: rs8679684: forward primer: 5′-CTC CTT TAT TTT GGG ATC TTG TGA A-3′, reverse primer: 5′-CCT GCT CCT TAT TCT GAG CTT AGA A-3′, probe specific for T allele: 5′-FAM-AGT GGT AAG TAT AGG ATT G-MGB-3′, probe specific for A allel: 5′-VIC-AGT GGT AAG TAA AGG AT-MGB-3′. Primers and probes for 19131AG SNP: forward primer: 5′-AGC AGG AAT GGG ACC TCA GAT-3′, reverse primer: 5′-GCA AAA GTA AAA GCA CTC TGA AGT CA-3′, probe for G allele: 5′-VIC-TGGTGCTAATGTCCT-MGB-3′ and for A allele: 5′-FAM-TGG TGC TAA TAT CCT-MGB-3′. Fluorescent signals were detected both real-time and after the PCR amplification, and were evaluated by the Sequence Detection Software 1.4. Allele frequencies were calculated for both breeds (and for the Hungarian and Belgian populations of Border Collies) separately. Hardy-Weinberg Equilibrium and Linkage Disequilibrium analyses were also carried out for the two breeds separately by Haploview 4.2 program [34].

### Results

**Figure 1:**
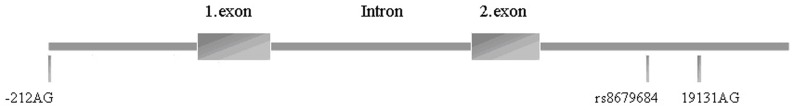
The three polymorphisms identified in the dog OXTR gene. The figure shows the canine OXTR gene with exons 1 & 2, the intron and the surrounding regulatory regions. Polymorphisms in the 5′ and 3′ UTR regions are marked with their rs number if applicable or with their position and base change.

By direct sequencing of protein coding and the surrounding regulatory untranslated regions of the dog OXTR gene one known (rs8679684) and two novel (−212AG, 19131AG) single nucleotide polymorphisms (SNP) were found. The −212AG SNP is located in the 5′ flanking region, whereas rs8679684 and 19131AG SNPs can be found in the 3′ untranslated region of the gene (**Figure 1**). The identified polymorphisms were subsequently genotyped in 104 German Shepherds and 103 Border Collies used in Study 1. Linkage analysis revealed that the rs8679684 and 19131AG SNPs are in strong linkage disequilibrium both in German Shepherds (*D*′ = 0.98, *R* ^2^ = 0.96) and in Border Collies (*D*′ = 1.0, *R* ^2^ = 1.0).

Allele frequencies of the two breeds are presented in **Table 4**. Hungarian and Belgian Border Collies did not differ in allele frequencies for any of the three investigated SNPs (19131AG: χ^2^_(2)_ = 5.181, p = 0.075, −212AG: χ^2^_(2)_ = 4.384, p = 0.112, and rs8679684: χ^2^_(2)_ = 4.121, p = 0.127). Both breeds were in Hardy-Weinberg equilibrium for all three polymorphisms (**Table 4** **.**).

**Table 4:** Allele frequencies for the two breeds studied.

	−212AG				rs8679684				19131AG			
	AA	AG	GG	HWE	AA	AT	TT	HWE	AA	AG	GG	HWE
German Shepherd	0.12	0.48	0.39	p = 0.876	0.38	0.47	0.15	p = 1.000	0.37	0.49	0.14	p = 0.749
Border Collie (Hungary)	0.10	0.31	0.47	p = 0.203	0.00	0.03	0.92	p = 1.000	0.00	0.03	0.97	p = 1.000
Border Collie (Belgium)	0.02	0.23	0.66	p = 1.000	0.02	0.11	0.80	p = 0.486	0.02	0.14	0.84	p = 0.601

The proportion of each genotype is provided for German Shepherds and the two populations (Hungary, Belgium) of Border Collies separately. Statistical tests for Hardy-Weinberg Equilibrium (HWE) are also provided.

## Study 3

### Method

Gene × behavior associations were tested by comparing the phenotype (along the four behavioral scales described in Study 1) between dogs with different genotypes (for the three OXTR SNPs described in Study 2). ANOVA or independent samples t-test was applied depending on the allele frequencies. The analyses were conducted for German Shepherds and Border Collies separately because in Study 1 we found significant difference in the behavior of the two breeds. Rare homozygote genotypes were grouped together with heterozygotes for the present analysis. As the expected effect sizes for the contribution of one SNP to a behavioral trait were relatively small, similarly to other (human) gene × behavior studies [e.g. 25], [27] the statistical tests were not corrected for multiple comparison.

### Results

**Figure 2:**
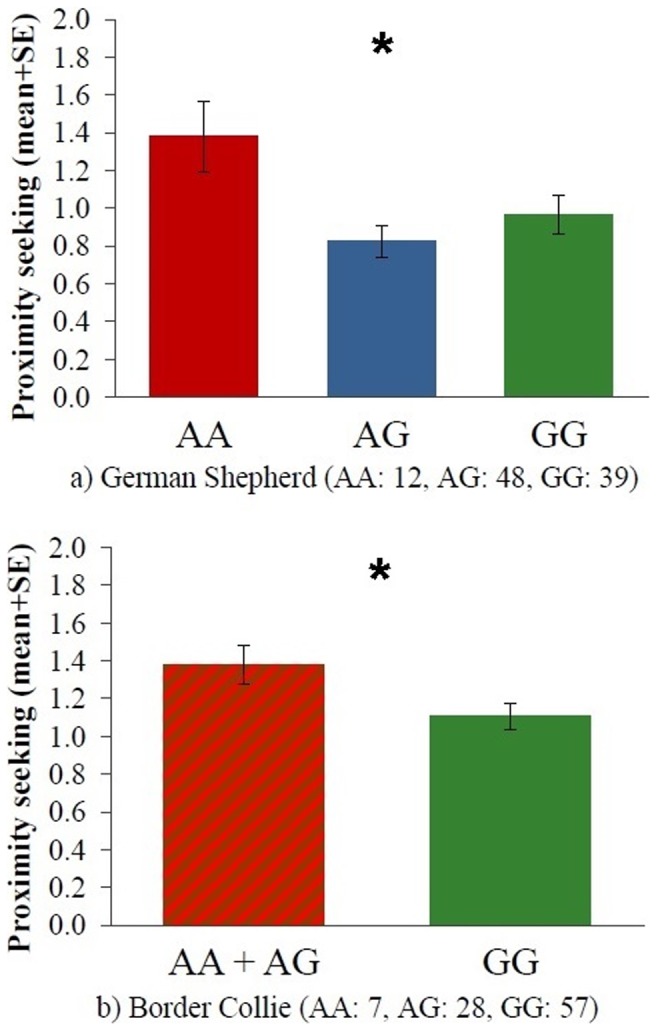
Proximity seeking scores mean differences between the different −212AG genotypes in German Shepherds (a) and Border Collies (b). Sample sizes for each genotype group are provided in parenthesis. *: p<0.05.

The −212AG polymorphism was associated with Proximity seeking both in case of German Shepherds (F = 4.030, p = 0.021) and Border Collies (t = 2.282, p = 0.025); carrying the G allele, was associated with lower proximity seeking in both breeds (**Figure 2**). Associations with the other three behavioral scales were not significant (all p>0.05, **Table 5**).

**Table 5:** Associations of the OXTR SNPs with the behavioral scales.

	German Shepherds			Border Collies		
	*−212AG*	*rs8679684*	*19131AG*	*−212AG*	*rs8679684*	*19131AG*
Proximity seeking	F = 4.030*	t = 0.641 ns.	t = 0.931 ns.	t = 2.282*	t = 1.119 ns.	t = 0.964 ns.
Reaction to separation from owner	F = 1.083 ns.	t = 0.096 ns.	t = 0.147 ns.	t = 1.581 ns.	t = 0.738 ns.	t = 0.473 ns.
Friendliness	F = 0.171 ns.	t = 2.570*	t = 2.724**	t = 0.739 ns.	t = 2.412*	t = 2.800*
Looking at humans	F = 0.710 ns.	t = 0.140 ns.	t = 0.022 ns.	t = 1.514 ns.	t = 1.242 ns.	t = 1.514 ns.

: p<0.01,

: p<0.05,

ns.: p>0.05.

**Figure 3:**
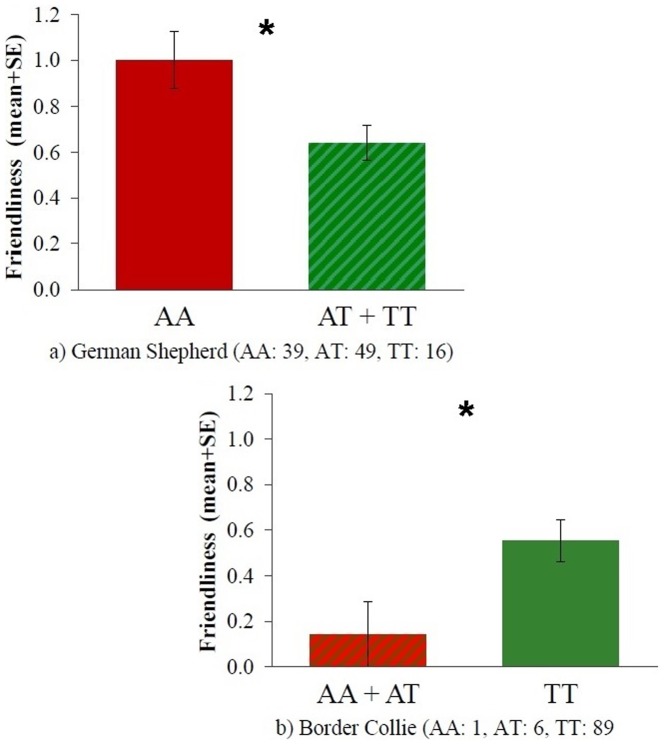
Friendliness scores mean differences between the different rs8679684 genotypes in German Shepherds (a) and Border Collies (b). *: p<0.05.

The rs8679684 polymorphism was associated with Friendliness both in case of German Shepherds (t = 2.570, p = 0.012) and Border Collies (t = 2.412, p = 0.033). However an opposite trend could be observed in the two breeds. In German Shepherds carriers of the A allele, as opposed to the T allele, achieved higher scores on the Friendliness scale, while in Border Collies individuals carrying the A allele were less friendly (**Figure 3**). Associations with the other three behavioral scales were not significant (all p>0.05, **Table 5**).

**Figure 4:**
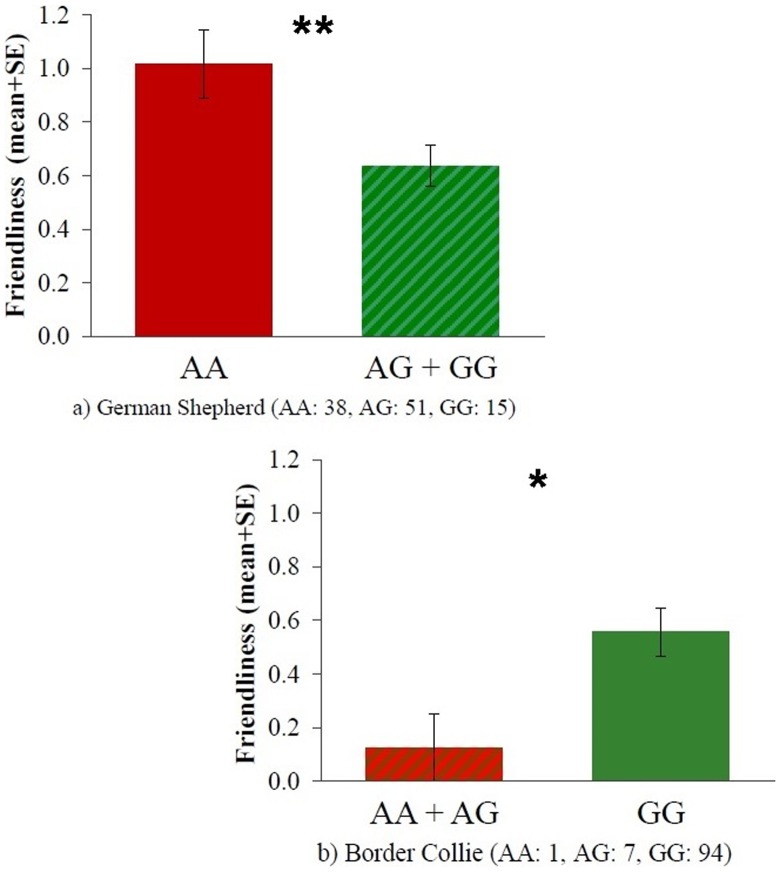
Friendliness scores mean differences between the different 19131AG genotypes in German Shepherds (a) and Border Collies (b). *: p<0.05, **: p<0.01.

As a result of linkage disequilibrium the 19131AG polymorphism, similarly to the rs8679684 SNP, was associated with Friendliness both in case of German Shepherds (t = 2.724, p = 0.008) and Border Collies (t = 2.800, p = 0.013). The presence of the A allele, as opposed to the G allele was associated with higher Friendliness scores in German Shepherds and lower Friendliness scores in Border Collies (**Figure 4**). Associations with the other three behavioral scales were not significant (all p>0.05, **Table 5**).

## Discussion

The main result of Study 1 is the development of a test series measuring social behavior in dogs, a prerequisite indispensable for genotype × phenotype analysis [35]. Previous research has already developed test series for studying behavioral genetic associations (e.g. activity-impulsivity: [11], [36]) stressing the importance of direct and precise phenotyping. In the present study we found that the behavioral scales developed on a group of German Shepherds were valid for two populations of Border Collies, as well. Furthermore, as expected based on previously described breed differences [37]–[39], we found that the social behavior of German Shepherds and Border Collies was different.

Study 2 has determined three SNPs in the dog OXTR gene, two of which were previously unknown. The possible functional effect of these polymorphisms is that one of these SNPs (−212AG) is located in a CpG island and thus may alter the methylation pattern of the promoter region and thereby change the mRNA expression level, as suggested by several studies [40]–[42]. The other two SNPs (rs8679684, 19131AG) in the 3′ UTR region might also influence the amount of the protein expressed by altering miRNA binding [43], [44]. Molecular functional studies were not performed, thus further experiments (e.g. gene expression profiles in dogs with different genotypes) should determine the effect of these SNPs at the cellular level.

In Study 3 we provide the first evidence that polymorphisms in the OXTR gene are related to human directed social behavior in dogs. Although we do not, at this point, have any information about the intermediary (cellular and molecular) mechanisms directly involved in the regulation of the behaviors we observed, the indirect gene × behavior connection we observed is in line with previous findings in humans [45], [46]. Thus these results further extend the role of the dog as a model species in behavior genetic research [29] by possibly aiding future research leading to the understanding and treatment of human social disorders. However we also note, that due to the low effect sizes that are characteristic of these gene × behavior studies [47], [48] our results need to be replicated before more specific conclusions can be drawn.

The oxytocin system is most often related to prosocial behaviors [49] and trust [50]. Although it is hard to draw a parallel between the behavioral measurements in the present study and for example the computerized trust games that are most often used with humans, our results are in accordance with previous findings as we found that polymorphisms in the OXTR gene had an effect on the Proximity seeking and Friendliness of dogs.

We should note, however, that the polymorphisms related to Friendliness had an opposite effect on German Shepherds and Border Collies, suggesting that other genetic and cellular mechanisms (unexplored in the present study) might play a role in the regulation of this behavior besides our candidate gene. Recent accounts in the human literature have cautioned about the individual (e.g. motivation or anxiety of the subjects) and conditional (e.g. contextual) differences in the effects of oxytocin on social behavior [51] suggesting that it would be erroneous to assume that oxytocin broadly and invariantly improves social cognition. Our results somewhat parallel these ideas in that we also found that in dogs the influence of a SNP in the OXTR gene on Friendliness towards humans is conditional to a breed effect.

Polymorphisms in the OXTR gene have been shown to be related to security/insecurity of mother-infant attachment in humans [26] that manifests in behavior such as approach and physical contact towards the caregiver in reunion episodes. Our results are in agreement with this finding as in dogs an OXTR gene polymorphism was related to Proximity seeking; on the other hand we did not find any effect on the Reaction to separation from the owner.

It has also been shown that oxytocin increases looking at the eye-region of faces in humans [52], and based on this finding one could expect a general relationship between the oxytocin system and looking at the eyes/face of humans. However other studies have questioned if such a relationship exists [53] and we also could not find any effect of OXTR gene polymorphisms on how much dogs look into the face of humans. We should note, however, that the test used in the present study consisted of problem solving situations when the dogs had a chance to look back at their owners and/or the experimenter, while in the human studies [52], [53] subjects were presented with computerized stimuli on a monitor. This latter setup has also been used with dogs [54], and in this case it has been found that intranasally administered oxytocin decreased looking at the eye region [54].

This is the first behavioral genetic evidence for OXTR's previously suggested [55] involvement in interspecific (dog-human) interactions. Further studies should replicate and extend these preliminary findings, as well as to reveal the mediating molecular mechanisms. As the oxytocin system has been implicated in several human neurological disorders [21], the present results – together with the fact that the dog is a natural model of complex human illnesses [13] – open up the possibility for future research of the genetic background of certain social disorders.
